# Can the Use of Bayesian Analysis Methods Correct for Incompleteness in Electronic Health Records Diagnosis Data? Development of a Novel Method Using Simulated and Real-Life Clinical Data

**DOI:** 10.3389/fpubh.2020.00054

**Published:** 2020-03-05

**Authors:** Elizabeth Ford, Philip Rooney, Peter Hurley, Seb Oliver, Stephen Bremner, Jackie Cassell

**Affiliations:** ^1^Department of Primary Care and Public Health, Brighton and Sussex Medical School, Brighton, United Kingdom; ^2^Department of Physics and Astronomy, University of Sussex, Brighton, United Kingdom

**Keywords:** electronic health records, patient data, data quality, missing data, Bayesian analysis, methodology

## Abstract

**Background:** Patient health information is collected routinely in electronic health records (EHRs) and used for research purposes, however, many health conditions are known to be under-diagnosed or under-recorded in EHRs. In research, missing diagnoses result in under-ascertainment of true cases, which attenuates estimated associations between variables and results in a bias toward the null. Bayesian approaches allow the specification of prior information to the model, such as the likely rates of missingness in the data. This paper describes a Bayesian analysis approach which aimed to reduce attenuation of associations in EHR studies focussed on conditions characterized by under-diagnosis.

**Methods:** Study 1: We created synthetic data, produced to mimic structured EHR data where diagnoses were under-recorded. We fitted logistic regression (LR) models with and without Bayesian priors representing rates of misclassification in the data. We examined the LR parameters estimated by models with and without priors. Study 2: We used EHR data from UK primary care in a case-control design with dementia as the outcome. We fitted LR models examining risk factors for dementia, with and without generic prior information on misclassification rates. We examined LR parameters estimated by models with and without the priors, and estimated classification accuracy using Area Under the Receiver Operating Characteristic.

**Results:** Study 1: In synthetic data, estimates of LR parameters were much closer to the true parameter values when Bayesian priors were added to the model; with no priors, parameters were substantially attenuated by under-diagnosis. Study 2: The Bayesian approach ran well on real life clinic data from UK primary care, with the addition of prior information increasing LR parameter values in all cases. In multivariate regression models, Bayesian methods showed no improvement in classification accuracy over traditional LR.

**Conclusions:** The Bayesian approach showed promise but had implementation challenges in real clinical data: prior information on rates of misclassification was difficult to find. Our simple model made a number of assumptions, such as diagnoses being missing at random. Further development is needed to integrate the method into studies using real-life EHR data. Our findings nevertheless highlight the importance of developing methods to address missing diagnoses in EHR data.

## Introduction

### Uses of Electronic Health Record Data for Epidemiology

The use of routinely collected data from patients' medical records has gained traction in epidemiology and health research in the last two decades. In many jurisdictions, patient data from electronic health records (EHRs) are stripped of identifiers and curated into large databases, and linked with other sources of health and administrative data, and thus used to gain insights into drug safety, disease risk factors, and to aid health service planning.

The United Kingdom (UK) has a rich history of using patient data from the National Health Service (NHS), which has coverage of almost the whole resident population and provides the opportunity for large, population-based datasets (1). Other countries which have nationalized healthcare systems, such as the Netherlands or Denmark, or which have large private providers, such as Kaiser Permanente, Mayo Clinic, or the Veteran's Association in the USA, also curate and re-purpose patient data for research. One UK example is the Clinical Practice Research Datalink (CPRD), which is an ongoing primary care database of anonymized medical records from general practitioners (GPs) in the United Kingdom (2), and has been the basis of 886 published papers in the last 5 years (3).

Many important epidemiological studies have been conducted using these population-based, routinely collected data. For example, the safety of the measles, mumps, and rubella vaccine has been studied (4), and the impact on pregnancy complications of legislative changes to make public spaces smoke free (5), among many studies on the safety of drugs in population usage (6).

In addition, much methodological work has been put into establishing the validity and data quality of large databases of routinely collected patient data, and especially into the quality of linkages between datasets (7–10).

### Dimensions of Health Information Quality in Electronic Health Records Research

Because information accrues in these records through the course of routine interactions between patients and clinicians, data quality is variable and hard to assess. Data quality can be understood by reference to several dimensions: completeness, uniqueness, timeliness, consistency, accuracy, and validity (11). Of particular interest for EHRs may be the data quality domains of completeness, validity, and accuracy (12–14).

Given that the EHR is an imperfect representation of the illness state of an individual, and that it is in fact a collection of working notes of a single or of various clinicians, it cannot be expected that it will represent a perfect record of every condition in the patient. A patient may have a condition, e.g., influenza, but may not visit the doctor, so a diagnosis for this condition would be missing from their record (we use “condition” to represent the state of illness in the patient, and “diagnosis” to indicate the record of the condition in the EHR; as well as the identification of the condition by the clinician). For a chronic condition, diagnosis may happen elsewhere in the healthcare system (i.e., in specialist clinics) and the diagnosis may not be added to the primary care record for some time. In some conditions a clinical diagnosis is somewhat equivocal or may become more certain over a number of consultations. Some conditions are stigmatized or distressing and doctors may be wary of communicating unpalatable diagnoses. These conditions may therefore be labeled using symptom rather than diagnostic codes (15, 16) or be recorded in clinical free text notes rather than using the clinical coding system (17). Examples of the above scenarios are mental health conditions, such as anxiety and depression, stigmatized neurological conditions such as dementia, and chronic conditions which may have a “silent” onset, such as diabetes (18, 19). Figures from a range of studies and two meta-analyses shown in Table 1, they show that estimated rates of under-diagnosis for dementia, depression and anxiety, average about 50% (20–35). Further assessments of completeness of EHR data, such as the review by Chan et al. (36), show that missingness of parameters such as blood pressure and smoking status can be as high as 38–51%, but are less likely to be missing in populations where these parameters are important for clinical care, such as a high risk cardiovascular disease cohort. Bhaskaran et al. (37, 38) showed that BMI measurements were missing for between one-third and two-thirds of patients, with an increase in completeness achieved over time between 1991 and 2011. However, length of registration per patient does not necessarily indicate an improvement in diagnosis capture over time (39).

**Table 1:** Sensitivity and specificity of GP recognition and recording of anxiety, depression, and dementia.

**Study**	**Disorder**	**Data source/setting**	***N* patients**	**GP recognition case definition**	**Reference standard**	**Sensitivity (coded evidence) (%)**	**Specificity (%)**
Janssen et al. (20)	Anxiety	Netherlands Study of Depression and Anxiety longitudinal cohort (21 family practices)	816	ICPC diagnosis codes, medication, referral or free text reference to anxiety from medical record	Screened with Kessler-10 and diagnosis made with Composite International Diagnostic Interview	16.5	97.2
Kroenke et al. (21)	Anxiety	15 US Primary Care Clinics	965	Receipt of treatment for anxiety (medications, counseling, or psychotherapy)	GAD-7 screening followed by structured psychiatric interview	59.0	–
Fernández et al. (22)	Anxiety	77 primary care centers in Catalonia, Spain (DASMAP study)	666	ICD or ICPC codes in the medical record	Structured Clinical Interview DSM IV	32.0	90.0
Sinnema et al. (23)	Anxiety or Depression	23 General Practices in the Netherlands	444	Free text terms or ICPC codes for anxiety or depression	Screening on Kessler 10	31.0	–
Wittchen et al. (24)	Depression Anxiety Both	558 primary care physicians in Germany	17,739	Doctor's clinical appraisal questionnaire	Diagnostic screening questionnaire	64.3 34.4 43.2	–
Kessler et al. (25)	Depression or Anxiety	1 General Practice in North Bristol, UK	179	GP medical records for diagnosis, treatment and referral	GHQ questionnaire followed by Clinical Interview Schedule	39.0	–
Joling et al. (26)	Depression	33 General Practitioners in Leiden and Amsterdam, Netherlands	816	Medical records: diagnostic codes, medication, referral and free text	Composite International Diagnostic Interview	43.0	94.4
Kendrick et al. (27)	Depression	7 general practices in Southampton, UK	694	GP rating on questionnaire, and patient records	Hospital Anxiety and Depression Scale	33.3	88.5
Wittchen et al. (28)	Depression	633 German primary care doctors	20421	Doctor's questionnaire	Depression Screening questionnaire	28.9	88.3
Cepoiu et al. (29)	Depression	Meta-analysis of 36 studies	>10,000	Chart review or Physician questionnaire	Various screening questionnaires and structured clinical interviews.	36.4 (pooled)	83.7 (pooled)
Connolly et al. (30)	Dementia	6 primary care trusts in Greater Manchester (351 general practices) in UK	253,477 (>65 years)	Dementia registers in GP records	National prevalence estimates from Medical Research Council: Cognitive Function Aging Study, MRC CFAS, 1998	45.4	–
Walker et al. (31)	Dementia	7,711 GP practices in England	n/a	Primary care disease registers of the QOF	National Health Service England's ‘Dementia Prevalence Calculator'	41.6	–
O'Connor et al. (32)	Dementia	Seven Group GP practices in Cambridge		GP rating of diagnosis	MMSE followed by diagnostic interview (CAMDEX)	58.0	22.0
Collerton et al. (33)	Dementia	2 primary care trusts in Newcastle and Tyneside, UK	1,024	General practice records	Questionnaires and health evaluation	46.6	–
Lithgow et al. (34)	Dementia	Nursing home residents in Glasgow, UK	422	Diagnosis written in care plan/GP record	Standardized MMSE	64.5	–
Lang et al. (35)	Dementia	Meta-analysis of 23 global studies (Europe, north America, Thailand, China)	43,446	Majority: Medical records	Screening tools or diagnostic interviews	38.3	–

Missingness in EHR data is a recognized problem and multiple solutions have been proposed. Wells et al. (40) proposed a helpful model for understanding two types of missingness in EHRs. Firstly “clearly missing structured data” are data such as missing test results, or parameters such as blood pressure or BMI, where patients are expected to have a value. Secondly, there is “missing = assumed negative” data, and it is this second type that we focus on here. Rather than being planned measurements or variables which have either been collected into structured fields, or are missing; entries of diagnoses, or medical history are made in the record over time on the basis of clinic visits and the patient's presentation as well as the decisions and thought processes of the clinician. This leads to the situation where if there is evidence in the record that a patient has a condition (i.e., a “diagnosis”), we can identify them as a case. However, if a record has diagnosis recorded, we cannot know if data are “negative” or “missing” for that condition. We generally treat patients with no diagnosis for a condition as “negative,” i.e., they do not have the condition, but they may, in a few cases, be “positive but unlabeled” (41), that is, having the condition but missing a diagnosis for it.

Several statistical methods have been developed to deal with the first type of missing data (empty structured fields), most notably multiple imputation with chained equations (42–47). However, these approaches do not allow discrimination between negative and missing in the second case of missing diagnoses.

For EHR research, patients who have a condition of interest need to be identified or defined as “cases” of that condition for inclusion in a study. We find that many case definitions in EHR research, such as for rheumatoid arthritis or types of dementia [e.g., (48, 49)] prioritize specificity over sensitivity. That is, they require several pieces of information about a condition to exist in the record within a set time-frame, before the researcher can be satisfied that the patient really is a “case” for the purposes of the study. Even patients with some evidence of a condition may be left out of the case group developed for the study. Thus patients included as “cases” in a study may not be fully representative of patients with the condition in general. The problem of missing cases (or false negatives) in a study is likely to be greater than of false positive cases. Case validation methods in EHR research have often investigated the positive predictive value of their case definition, that is, how many patients identified as having a condition truly have that condition. They have rarely investigated how many patients with a condition are missed by their method of identifying cases (12). Additionally, it is extremely hard to determine sensitivity or specificity of case definitions in a large EHR database, because establishing a “ground truth” or gold standard to verify cases against is a costly process, usually involving sending questionnaires to the originating GP to validate information in the record.

It is widely recognized that misclassifications of patients due to missing or false diagnosis codes will impact on any use of primary care records for prevalence and incidence studies, resulting in incorrect estimates of the burden of disease in the population [e.g., (19)]. However, missed cases are much less widely discussed as an issue in studies looking to estimate associations between two conditions or between an exposure and outcome. The impact of a substantial proportion of cases being missed, when the association between two conditions is studied, has been recognized for decades as independent non-differential measurement error (50). It is known that this error is likely to attenuate associations and reduce the power of statistical tests to find associations, thus biasing results toward the null hypothesis. Clinically, this may impede our understanding of risk factors for a condition, or of drug side effects, for example. A worked example using conditional probabilities to show attenuation of estimated associations when diagnoses are missing, is given as a learning exercise in Box 1.

Box 1 **Worked example of conditional probabilities in misclassified cases showing attenuation of estimated associations when diagnoses are missing.**

We explore a simple hypothetical model in which patients might have two conditions, A and B. We work this model through using Bayesian nomenclature. Let us assume that the probability of a patient having condition A, if they have condition B, is 0.6

- P(A | B) = 0.6

and let the probability of a patient having condition A, if they do not have B, be 0.2.

- P(A | ¬B) = 0.2

These two conditions are imperfectly captured in the patient record. Let the probability of the patient having a diagnosis recorded for condition A, if they have A, be 0.6.

- P(D_A_ | A) = 0.6

There are also some false positives; let the probability of a patient without condition A nevertheless having a recorded diagnosis for A be 0.05.

- P(D_A_ | ¬A) = 0.05

Let condition B be slightly better captured in the EHR, so that the respective probabilities are 0.85 and 0.03.

- P(D_B_) | B) = 0.85
- P(D_B_ | ¬B) = 0.03

Let the prevalence, or overall probability of any patient having condition B, be 0.1.

- P(B) = 0.1

Suppose that we do not know the association between the two conditions, P(A | B), and we want to estimate this using the recorded diagnoses. A naïve approach will instead estimate the probability of having a recorded diagnosis of A, given a recorded diagnosis of B, thus: P(D_A_ | D_B_). We can demonstrate that the association given by P(D_A_ | D_B_) is not a good approximation of the association P(A | B).

The conditional probability P(D_A_ | D_B_) can be represented in terms of the joint probability of D _A_ and D_B_ and P(D_B_):

$$\begin{array}{l} P\left(D_{A}|D_{B}\right)=\;\frac{P\left(D_{A},D_{B}\right)}{P\;\left(D_{B}\right)} \end{array}$$

The joint probability is the total probability of each of the four ways of obtaining a diagnosis of A and B.

P(D_A_,D_B_) = P(A | B) · P(B) · P(D_A_ | A) · P(D_B_ | B) +

    P(A | ¬B) · P(¬B) · P(D_A_ | A) · P(D_B_ | ¬B) +

    P(¬A | B) · P(B) · P(D_A_ | ¬A) · P(D_B_ | B) +

    P(¬A | ¬B) · P(¬B) · P(D_A_ | ¬A) · P(D_B_ | ¬B)

and

    P(D_B_) = P(B) · P(D_B_ | B) + P(¬B) · P(D_B_ | ¬B)

In our hypothetical scenario these can be evaluated as

P(D_A_,D_B_) = (0.6 × 0.1 × 0.6 × 0.85) + (0.2 × 0.9 × 0.6 × 0.03) + (0.4 × 0.1 × 0.05 × 0.85) + (0.8 × 0.9 × 0.05x0.03) = 0.03663; and

P(D_B_) = (0.1x0.85) + (0.9x0.03) = 0.112

Thus, from recorded cases only, we estimate the association between A and B is

- P(D_A_ | D_B_) = 0.03663/0.112 = 0.33.

Note that the true association, given at the beginning of this section, is P(A | B) = 0.6. Thus we have demonstrated that assuming P(D_A_ | D_B_) = P(A | B) can lead to attenuated estimations of association.

Given the evidence that many conditions are under-recorded in EHR data, that case definitions are not perfectly sensitive, and that these factors are likely to attenuate associations within analyses, we aimed to develop a method which could reduce the effect of independent non-differential measurement error on estimates of associations in EHR data.

### Using Bayes' Theorem to Address This Attenuation in EHR Data Analyses

Bayes' theorem is a rigorous method for interpreting evidence in the context of previous knowledge or experience (51). Bayes' theorem describes how to update our understanding of the probability of events given new evidence.

Given a hypothesis, H, and evidence (E; that is, data collected in a study), Bayes' theorem states that the relationship between the prior probability of the hypothesis being true before obtaining the evidence, P(H), and the probability of the hypothesis being true given the evidence, P(H|E), is as follows:

$$\begin{array}{l} \mathrm{Pr}\left(H|E\right)=\;\frac{\mathrm{Pr}\left(E|H\right)\mathrm{Pr}\left(H\right)}{\mathrm{Pr}\left(E\right)} \end{array}$$

Bayes' theorem could be used with EHR data to inform any statistical model of likely misclassification rates, using prior information. We propose using a Bayesian framework for estimating the associations between conditions, within which prior estimates of the likely misclassification rates, both positive and negative, can be included. Our approach is to use this information to account for the misclassifications in the data, with the hypothesis that this will generate estimates of associations closer to real values. As this is a novel approach, we aimed to develop a first, simple, proof of concept model using the Bayesian approach. With EHR data, we only have the recorded diagnosis status of patients. This is not a perfect reflection of true condition status across the whole population and we cannot know patients' true condition status from the EHR database. Thus, to develop and assess our method, we generated synthetic data, and ran simulations (Study 1). We then tried the approach in real life clinic data (Study 2).

## Study 1: Simulations Methods and Results

### Dataset

We created synthetic datasets approximating simple structured EHR data, so that each patient would have a known true condition status and a recorded diagnostic status for a small number of conditions, for which there was a known rate of misclassification. Each synthesized patient therefore had two layers of data; their true condition status (which would be unknown in real clinic data), and their recorded diagnostic status (as reported in clinic data). We created these data for three related conditions, A, B, and C. The three conditions were related in a generalized linear model relationship where the probability of the true status of condition A was determined by whether the patient truly had conditions B and C using the formula A = β_0_(intercept) + β_1_(B) + β_2_(C). Four sets of values of β_0_, β_1_, and β_2_ were chosen to represent a wide range of associations and are shown in Table 2.

**Table 2:** Parameters determining relationship between three conditions in synthetic datasets.

**Simulation**	**β_0_**	**β_1_**	**β_2_**
1	0.2	4	3
2	0.5	8	3
3	0.1	0.5	2.6
4	0.1	0.6	0.8

The rates of misclassification (the rate of mismatch between the true and the recorded condition status) assigned to each condition are shown in Table 3. These probabilities then determined whether each patient obtained a recorded diagnosis for their condition or not. Simulations additionally varied by number of patients within the dataset (100, 500, 1,000, 5,000, 10,000, and 20,000), giving a total of 24 synthetic datasets (4 sets of parameters × 6 sizes).

**Table 3:** Rates of misclassification in synthetic data, expressed as a set of parameters determining the conditional probabilities (see Appendix 1 in Supplementary Material for terminology).

**Parameter**	**Value**
P(DA | A)	0.86
P(DA | ~A)	0.02
P(DB | B)	0.65
P(DB | ~B)	0.08
P(DC | C)	0.68
P(DC | ~C)	0.15

### Analysis Method

Our objective was to estimate associations between three conditions of A, B, and C in this synthetic data, using a conventional generalized linear model (GLM) and Bayesian modeling approximating GLM, and explore the relative accuracy of the two approaches in estimating parameters β_0_, β_1_, and β_2_.

We determined the association between the variables only from the recorded diagnosis status. As this was mostly under- rather than over-diagnosed in our synthetic data, we expected an attenuation of associations compared to the real association in the true condition status. We analyzed the datasets using the software JAGS, a Gibbs Sampler (52), which is a program for the statistical analysis of Bayesian hierarchical models by the Markov Chain Monte Carlo method. It allowed us to fit both a traditional logistic regression (LR) model and a Bayesian logistic regression model, in which the rates of misclassification were introduced to the model as Bayesian priors (model specification and terminology is given in Appendix 1 in Supplementary Material).

The role of the two types of logistic regression models was to try to recover the parameters which determined the true relationships between the three conditions (β_0_, β_1_, and β_2_). Parameters were estimated with 95% confidence intervals (CIs) in the LR and 95% credibility intervals in the Bayesian models.

### Results

Both types of model (LR and Bayesian LR) ran successfully and converged, and produced estimates for the parameters. The 95% confidence or credibility intervals narrowed as more patients were included in the simulation, as would be expected (range 100–20,000).

**Figure 1:**
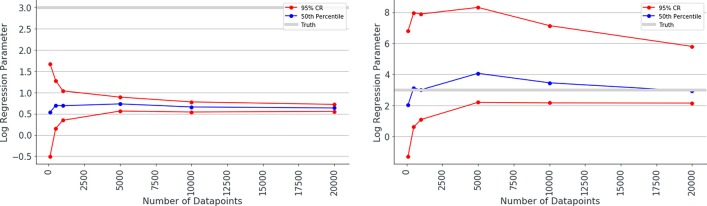
Median estimated value and 95% confidence intervals for one of the parameters (β_2_ in simulation 1, shown on y axis) in a simulation. Estimated value is plotted against number of data points used to make the fit (x axis), when misclassification rates were not modeled (left) and were modeled (right) as Bayesian priors. Notice the true value for parameter β_2_ is shown as a gray line and has the value 3.0. The traditional logistic regression substantially underestimates the association, whereas the credibility intervals of the Bayesian logistic regression are substantially wider but span the correct value.

**Figure 2:**
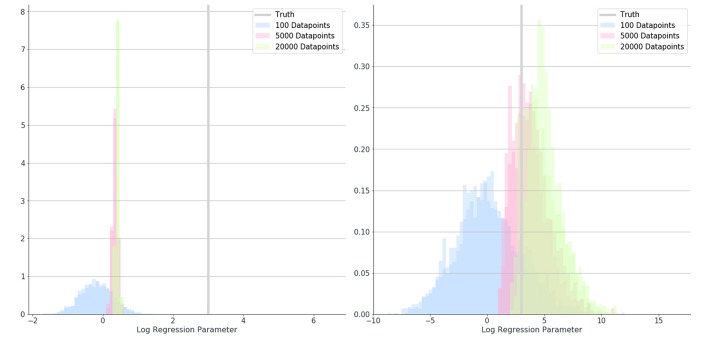
Posterior distributions for the estimate of a parameter (β_2_) in simulation 1 where 100 (blue), 5,000 (red), and 20,000 (green) data-points were used. Distributions show results when misclassification rates were not modeled (left) and when they were (right). The true value for parameter B is shown as a gray bar (β_2_ = 3).

Overall, the logistic regression models produced estimates for the parameters which were smaller than the true parameters (attenuation) and had narrow 95% confidence intervals giving the impression that estimations were very accurate, however, CIs only overlapped the true parameter in simulations with small associations and small numbers of patients. The Bayesian logistic regression models produced parameter estimates that overlapped the true parameter value in all but one case, although with wider credibility intervals. These effects can be seen in the exemplar graphs Figures 1, 2. The full results of 24 simulations are given in Appendix 2 (Supplementary Material).

### Discussion of Simulation Study

These simulation studies provide a proof of concept that by adding in known information about population-based misclassification rates as Bayesian priors to conventional analyses, we can reduce the attenuation in estimations of associations between conditions.

The simulations also show that in the analyses using Bayesian priors to model misclassification rate, the credibility intervals around the parameter estimations were much wider than in conventional analyses, and that in almost all simulations these wider credibility intervals spanned the correct parameter value. Although these confidence intervals were much wider than in conventional analyses, they narrowed as more patients were added to the analysis, and were still narrowing at 20,000 patients. This demonstrates that with the Bayesian approach, it may be more important to have larger datasets for achieving precise estimations of associations.

## Study 2: EHR Data Methods and Results

Following these simulations, we then undertook to investigate whether this Bayesian approach may improve our ability to make predictions about which patients are developing dementia, using data from anonymized patient records from UK primary care. It is estimated from a range of different studies that about one-third of people living with dementia do not get a diagnosis (53). Additionally, several associated conditions which may act as predictors of dementia, including depression, anxiety, and diabetes, are known to be under-diagnosed in primary care. Thus, many associations between variables in our model may be attenuated due to misclassification.

### Dataset

We used data from the UK Clinical Practice Research Datalink (CPRD) (2). We used a case-control design. Dementia cases were selected from the CPRD database if they were over 65 years and had one or more dementia code in their record and the first of these was recorded between 2000 and 2012, if they came from a practice who had met acceptable quality standards, and if they had a minimum of 3 years of up-to-standard quality data prior to the first dementia code. We used 1-to-1 matching of control cases by age, sex and GP practice; controls were required to have no dementia codes anywhere in their record, and controls who had evidence of dementia in the form of Alzheimer's specific medication prescriptions or “dementia annual review” codes were removed from the sample. The entire patient record prior to the first dementia code, or a matched date in the controls, was extracted (total *N* = 93,120), but we analyzed only data from the 5 years preceding diagnosis or matched date in controls. We drew up code lists representing 70 potential variables which our research suggested might be predictive of the condition (54). We matched these to clinical codes found in the patient clinical, referral, test and immunization sections of the patient records. Full details of the sample and the variables in the analysis can be found elsewhere (55) and the full list of variables is given in Appendix 3 (Supplementary Material).

### Analysis Methods

We used LASSO penalized logistic regression (LR) (56) to combine and select a minimum set of best predictors from the 70 added to the model, with dementia status as the binary outcome. We aimed to create models which best discriminated between cases and controls, using a random cut of 80% of the data as a training set and 20% as a validation set. We assessed estimates of association (LR coefficients) between each variable and the outcome. We also assessed the ability of the model to correctly classify cases and controls using the Area Under the Receiver Operating Characteristic Curve (AUROC), which plots false positive rate against true positive rate for every possible threshold of the model.

We specified three methods, an LR with no Bayesian priors, and two LRs, in which we used generic estimates of misclassification for all variables, again using the software JAGS (SourceFourge). We modeled low misclassification rates (P(D(cond)|cond) = 0.95; P(D(cond)|¬cond) = 0.015) in one analysis and high misclassification rates (P(D(cond)|cond) = 0.85; P(D(cond)|¬cond) = 0.04) in the second analysis.

### Results

**Figure 3:**
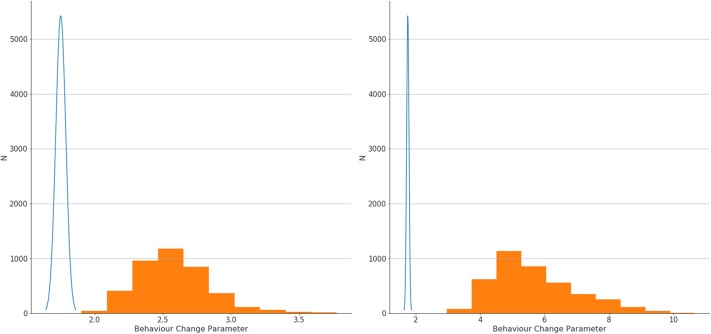
Estimation of confidence intervals of LR coefficients for association between behavior change and dementia with no errors modeled (blue line) and with small errors modeled (left graph, orange blocks) and large errors (right graph, orange blocks).

The logistic regression model produced small LR coefficients for all predictors, and the addition of Bayesian priors in the models resulted in higher LR coefficients. There was a small increase in parameters with the small errors modeled, and a larger increase if larger errors were modeled. Results showing LR parameters for the top 20 predictors in the model are found in Table 4. If we look at the highest ranked predictor of recorded Behavior Change, the estimate of association between this and dementia went up substantially from 1.75 with no errors modeled, to 2.56 when small errors were modeled, to 5.40 when large errors in classification were assumed. This exemplar variable is shown in Figure 3.

**Table 4:** Change in Logistic Regression (LR) coefficients when misclassification errors were modeled as Bayesian priors.

**Condition (Yes vs. No)**	**LR coefficient**	**LR Bayes small errors**	**LR Bayes large errors**
Behavior change	1.75	2.56	5.4
Third party consultation	0.65	0.82	1.43
Depression	0.58	0.72	1.21
Possible falls	0.41	0.51	0.81
GP home visit	0.40	0.42	0.67
Did not attend	0.36	0.43	0.67
Stroke	0.33	0.46	0.79
Cerebrovascular disease	0.26	0.25	0.41
Receives home care	0.18	0.24	0.41
Attended emergency room	0.18	0.22	0.36
Anxiety	0.18	0.18	0.32
Depressive symptoms	0.14	0.22	0.49
Constipation	0.09	0.13	0.23
Lower limb fracture	0.01	−0.001	0.006
Urinary tract infection	−0.02	0.03	0.06
Impaired mobility	−0.03	−0.003	0.05
Non-urgent hospital admission	−0.03	−0.03	−0.03
Social services involvement	−0.13	−0.21	−0.41
Living in a nursing home	−0.14	−0.14	−0.2
(Intercept)	−0.72	−0.82	−1.16

When predictors were combined in a multivariable model and the accuracy of the model to classify cases and controls assessed using Area Under the Receiver Operating Characteristic Curve (AUROC), we found no improvement in the accuracy of prediction in the overall model by the addition of Bayesian priors. Whether or not we used Bayesian priors in the Logistic Regression model, our model resulted in exactly the same AUROC (not pictured as the curves exactly overlaid).

### Discussion of EHR Data Analysis

Our initial analyses suggest that the Bayesian method, of modeling misclassification rates as priors, works in the same way on real clinic data as it did with synthetic data. The introduction of Bayesian priors appears to increase estimates of association and increase the width of confidence intervals around these estimates. The use of generic rather than condition-specific priors, did not result in any improvement in accuracy of classification in a multivariable model, which is a limitation of our approach that we explain below. However, we noted a range of implementation challenges when applying this approach to real clinic data, which we outline in the next section.

## Discussion

Simulation studies confirmed a substantial problem of attenuation of estimates of association when diagnoses are missing or patients are misclassified in EHR data. We have identified an approach which shows promise for dealing with this attenuation in EHR data. This method was simple to specify, and in simulated data, which mimicked misclassification in EHR data, we were able to recover the true associations between variables. We found that a traditional logistic regression model gave estimates which were attenuated compared to true associations between simulated conditions, yet our novel Bayesian method was able to estimate correctly the true association. However, after trialing the approach on real clinical data, we note a number of challenges to implementing our approach, which must be addressed before it can be adopted widely by researchers using EHR data.

### Identifying the Correct Priors for Real Life EHR Data

The first implementation challenge we encountered using real life clinic data was identifying what the values of the priors should be. In certain conditions (dementia, anxiety, depression, diabetes), the rates of under- and possibly over-diagnosis have previously been examined and estimated. However, rates of misclassifications have simply not been studied in a range of other conditions, such as stroke, coronary heart disease, or fractures. Additionally, there is no clear way of establishing under-recording or misclassification for more social, behavioral, or contextual variables such as third party consultations or receipt of care in the home, for which there is unlikely to be any objective measure against which to validate recording. Thus, establishing valuable prior information about likely rates of misclassification proved extremely difficult in real clinical data. We took the approach of using generic rates of errors across all variables, which limited our understanding of the potential of our method, as common sense understanding would suggest that the error rates must be different in different conditions. These findings should serve as a motivation to clinicians, epidemiologists, and data scientists to find ways to obtain this important and currently missing information. Sources of linked clinical data such as linked primary care and hospital data, or research cohort data linked to medical records, would be invaluable for quantifying missingness in EHRs for various conditions in a more automated way than manual chart review or GP surveys. Several studies have utilized this method to understand quality of recording in various clinical sources, e.g., Herrett et al. (57) for acute myocardial infarction and De Lusignan et al. (58) for osteoporosis. Using this method of two or more linked sources of data has multiple advantages; it allows for reducing “missing = assumed negative” in the database by triangulation from various sources, it would give indications of the likely rate of missingness in any one source, and it informs investigations on how missingness on one variable might affect missingness on another. Even where misclassifications cannot accurately be determined due to lack of a gold standard source of data, some knowledge of the levels of missingness would still allow analysts to include prior misclassification as a distribution rather than a fixed value in an analysis model.

### Validating the Change in Estimations

The second implementation challenge with this approach in real clinic data is that we have no accessible way of establishing a ground truth to validate the change in estimations of associations achieved by the introduction of Bayesian priors. Thus, we cannot know if these changes in estimates represent now the true association between the two conditions under study. However, it would be equally true to say that there is no way to validate that traditional approaches give the right answers, and we have shown that traditional approaches will only give the right answer if there are no misclassifications in the dataset.

### Assumptions Made Within This Approach

A third challenge for future development is to build a more complex model that more closely represents the causes of missingness in real life. Our approach was a simple, first proof of concept, and as currently specified assumes independence of reasons for missingness between different variables, or rather, that diagnoses are missing at random. However, we know that reasons for missingness or misclassification on one variable are likely to be related to reasons for missingness of another variable. Conversely, if a patient has a symptom a doctor may send a patient for a range of tests which result in several diagnoses simultaneously. The impact of this questionable assumption on the results obtained by the model is not currently clear, and should be explored in further simulation studies. The model also assumes that patients with a condition who receive a diagnosis do not differ systematically from those patients with a condition who do not have a diagnosis recorded. Again this is an implausible assumption, and the Bayesian priors that we specified do not attempt account for any systematic differences between these two groups. However, for all analysis methods which attempt to deal with complexity and quality in real life data, there is a tension between starting with a simple model which has a chance of converging, and a model which can be more true to life, but very complex, and which researchers cannot agree the granular parameters for. We aimed to achieve proof of concept here, and acknowledge that the approach can be further developed in time.

### No Improvement in Model Classification of Cases and Controls (AUROC)

In real life clinical data, where we added uniform Bayesian priors for all predictors, we did not see an improvement in the model's ability to discriminate between cases and controls. This is because the ROC curve analysis effectively uses the rank of the participants in order of their probability of having a positive rather than negative outcome in the classification analysis (59). With uniform rates of misclassification applied across predictors, these rankings did not change, despite higher estimates of association. This can be seen in Table 4, where variables, on the whole, did not swap in precedence despite increasing estimates of LR parameters. With more accurate and individualized estimates of misclassification, tailored to each predictor or condition, we would expect to see differences on ROC curves for the Bayesian analysis compared to traditional analyses. The ROC curve analysis also gives insight into which types of data analysis might be most affected by misclassification in the dataset. A simple estimation of association between symptom and condition, or exposure and outcome, may be highly affected. However, in a classification analysis, examined by a ROC curve, the ranking of which patients in the dataset are more likely to have a condition might be unchanged by missingness, except perhaps where missingness is associated also with the likelihood of having the condition.

### Summary and Conclusions

In summary, we have demonstrated that many conditions are misclassified or missing in EHR data, because, due to the way they are created, EHRs are an imperfect representation of the true status of health or illness in the individual. These errors in recording result in misclassification of cases when data from EHRs are used in research studies. In studies estimating the association between two variables in EHRs, this misclassification, which is more likely to involve missed cases than false positive cases, results in an attenuation of estimates of association between the two variables under study and a bias toward the null. We have shown how this attenuation can be ameliorated by using Bayesian priors in a Bayesian logistic regression paradigm in which population rates of misclassification errors are modeled. We trialed this in a range of simulations and on real clinic data from UK primary care. However, we noted implementation challenges with rolling this approach out on real clinic data, most of which stemmed from the fact that at the present time, identifying true misclassification rates for different conditions is difficult. We note further that validating the change in estimates achieved with the modeled priors is challenging. Our simplistic, proof of concept model assumed that diagnoses are missing at random, models which allow for more complexity should be developed for future work. Additionally, we found modeling of generic misclassification errors made little difference to overall predictive performance, assessed by AUROC, in a multivariable model. Future work should investigate whether error rates individualized to conditions can lead to improvements in the accuracy of model discrimination.

We have shown that missingness and incompleteness of diagnosis data within EHRs are important and overlooked issues in health information quality, can have a substantial impact on study results, and that analysis techniques should be developed to address these. Our approach to dealing with misclassified diagnoses in EHR data is novel and can be operationalized fairly simply using a Bayesian approach to logistic regression analysis. For full implementation, the research field will need to identify the misclassification rates in health data for a range of conditions, and in a range of healthcare settings. We hope the approach outlined in this paper will start a conversation within the EHR research community about how these key data quality issues can be tackled.

## Data Availability Statement

The datasets generated for this study will not be made publicly available. Synthetic datasets are available on application to the authors. The patient data that support the findings of this study are available from Clinical Practice Research Datalink (CPRD; www.cprd.com) but restrictions apply to the availability of these data, which were used under license for the current study, and so are not publicly available. For re-using these data, an application must be made directly to CPRD.

## Ethics Statement

This study was approved by the Independent Scientific Advisory Committee at the Medicines and Healthcare Products Regulatory Authority, UK, protocol number 15_111_R. Written informed consent for participation was not required for this study in accordance with the national legislation and the institutional requirements.

## Author Contributions

EF, SO, PR, and PH conceived and directed the study. PR created the synthetic data, managed the datasets, and conducted the analyses. PH, SO, and SB gave data analysis and interpretation advice. JC gave clinical advice for study 2. EF wrote the manuscript. All authors provided critical feedback on the manuscript and approved the final version.

### Conflict of Interest

The authors declare that the research was conducted in the absence of any commercial or financial relationships that could be construed as a potential conflict of interest.
