# Supplementary material for: Can the Use of Bayesian Analysis Methods Correct for Incompleteness in Electronic Health Records Diagnosis Data? Development of a Novel Method Using Simulated and Real-Life Clinical Data
Source: Front Public Health. 2020 Mar 5;8:54. doi: 10.3389/fpubh.2020.00054 (PMC7066995; doi:10.3389/fpubh.2020.00054)
Supplement: Supplementary file 1 [file Table_1.DOCX]

**Appendix 1: Model Specification**

**Logistic Regression Model Without Errors**

Diag(A[i]) ~ Bernoulli(P(A[i])

Diag(B[i]) ~ Bernoulli(P(B))

Diag(C[i]) ~ Bernoulli(P(C))

logit(P(A[i)]) <- β_0_ + β_1_*Diag(B[i]) + β_2_*Diag(C[i])

β_0_ ~ Normal(0,100)

β_1_ ~ Normal(0,100)

β_2_ ~ Normal(0,100)

**Logistic Regression Model With Errors**

True_B[i] ~ Bernoulli(P(B | Diag(¬B)) + (P(B | Diag(B))- P(B | Diag(¬B)))*Diag(B[i]))

True_C[i] ~ Bernoulli(P(C | Diag(¬C)) + (P(C | Diag(C))- P(C | Diag(¬C)))*Diag(C[i])

Diag(A[i])~ Bernoulli(P(Diag(A) | ¬A) + (P(Diag(A) | A)- P(Diag(A) | ¬A))*True_A[i])

True_A[i] ~ Bernoulli(Exp(β_0_ + β_1_*True_B[i] + β_2_*True_C[i]) / (1+Exp(β_0_ + β_1_*True_B[i] + β_2_*True_C[i])))

β_0_ ~ Normal(0,100)

β_1_ ~ Normal(0,100)

β_2_ ~ Normal(0,100)

β_0_ ~ Normal(0,100)

β_1_ ~ Normal(0,100)

β_2_ ~ Normal(0,100)

**Terminology**

True_Condition[i] signifies if patient “i” has Condition (i.e. equals 1), or not (i.e. equals 0)

X ~ Bernoulli(Y) signifies that X was drawn from a Bernoulli distribution with probability of success Y.

P(Condition) is the probability of having a condition.

P(Condition | Diag(Condition)) is the probability of having a condition, given they have a diagnosis for that condition.

Diag(B[i]) signifies if patient “i” has a diagnosis for Condition (i.e. equals 1), or not (i.e. equals 0)

β_0_, β_1_ and β_2_ are the logistic regression parameters.

¬A is equivalent to “not A”

β_0_ ~ Normal(0,100) shows that A has a weakly informative prior that it was drawn from a normal distribution with mean 0, and sigma 100.
