# Supplementary material for: Can the Use of Bayesian Analysis Methods Correct for Incompleteness in Electronic Health Records Diagnosis Data? Development of a Novel Method Using Simulated and Real-Life Clinical Data
Source: Front Public Health. 2020 Mar 5;8:54. doi: 10.3389/fpubh.2020.00054 (PMC7066995; doi:10.3389/fpubh.2020.00054)
Supplement: Supplementary file 2 [file Table_2.DOCX]

**Appendix 2**

**Model 1; 100 Patients**

| Fitting Technique | Parameter | 95% Confidence/Credibility Range (Mean) | True Value |
| --- | --- | --- | --- |
| Gibbs Sampler No Errors | A | -0.34-0.67 (0.16) | 0.2 |
| Gibbs Sampler No Errors | B | -0.72-1.07 (0.16) | 4 |
| Gibbs Sampler No Errors | C | -0.28-1.97 (0.76) | 3 |
| Gibbs Sampler With Errors | A | -0.25-1.15 (0.39) | 0.2 |
| Gibbs Sampler With Errors | B | -3.57-6.85 (1.48) | 4 |
| Gibbs Sampler With Errors | C | -0.47-7.41 (2.62) | 3 |

**Model 2; 100 Patients**

| Fitting Technique | Parameter | 95% Confidence/Credibility Range (Mean) | True Value |
| --- | --- | --- | --- |
| Gibbs Sampler No Errors | A | -0.03-1.01 (0.50) | 0.5 |
| Gibbs Sampler No Errors | B | -1.03-0.73 (-0.17) | 8 |
| Gibbs Sampler No Errors | C | -0.53-1.73 (0.54) | 3 |
| Gibbs Sampler With Errors | A | 0.17-1.91 (0.87) | 0.5 |
| Gibbs Sampler With Errors | B | -4.62-6.32 (0.15) | 8 |
| Gibbs Sampler With Errors | C | -1.11-7.10 (1.97) | 3 |

**Model 3; 100 Patients**

| Fitting Technique | Parameter | 95% Confidence/Credibility Range (Mean) | True Value |
| --- | --- | --- | --- |
| Gibbs Sampler No Errors | A | -0.57-0.44 (-0.08) | 0.1 |
| Gibbs Sampler No Errors | B | -0.64-1.15 (0.24) | 0.5 |
| Gibbs Sampler No Errors | C | -0.30-1.93 (0.76) | 2.6 |
| Gibbs Sampler With Errors | A | -0.62-0.89 (0.10) | 0.1 |
| Gibbs Sampler With Errors | B | -4.37-7.30 (1.48) | 0.5 |
| Gibbs Sampler With Errors | C | -0.36-6.95 (2.44) | 2.6 |

**Model 4; 100 Patients**

| Fitting Technique | Parameter | 95% Confidence/Credibility Range (Mean) | True Value |
| --- | --- | --- | --- |
| Gibbs Sampler No Errors | A | -0.55-0.45 (-0.04) | 0.1 |
| Gibbs Sampler No Errors | B | -0.84-0.89 (0.00) | 0.6 |
| Gibbs Sampler No Errors | C | -0.68-1.35 (0.28) | 0.8 |
| Gibbs Sampler With Errors | A | -0.63-0.97 (0.11) | 0.1 |
| Gibbs Sampler With Errors | B | -5.16-6.30 (0.54) | 0.6 |
| Gibbs Sampler With Errors | C | -1.70-6.68 (1.42) | 0.8 |

**Model 1; 500 Patients**

| Fitting Technique | Parameter | 95% Confidence/Credibility Range (Mean) | True Value |
| --- | --- | --- | --- |
| Gibbs Sampler No Errors | A | 0.03-0.45 (0.23) | 0.2 |
| Gibbs Sampler No Errors | B | -0.18-0.72 (0.27) | 4 |
| Gibbs Sampler No Errors | C | 0.15-1.25 (0.68) | 3 |
| Gibbs Sampler With Errors | A | 0.11-0.72 (0.40) | 0.2 |
| Gibbs Sampler With Errors | B | -0.21-7.38 (2.46) | 4 |
| Gibbs Sampler With Errors | C | 0.56-7.54 (2.76) | 3 |

**Model 2; 500 Patients**

| Fitting Technique | Parameter | 95% Confidence/Credibility Range (Mean) | True Value |
| --- | --- | --- | --- |
| Gibbs Sampler No Errors | A | 0.14-0.57 (0.35) | 0.5 |
| Gibbs Sampler No Errors | B | -0.13-0.73 (0.30) | 8 |
| Gibbs Sampler No Errors | C | 0.13-1.26 (0.69) | 3 |
| Gibbs Sampler With Errors | A | 0.31-0.90 (0.60) | 0.5 |
| Gibbs Sampler With Errors | B | -0.06-7.27 (2.49) | 8 |
| Gibbs Sampler With Errors | C | 0.58-7.56 (3.04) | 3 |

**Model 3; 500 Patients**

| Fitting Technique | Parameter | 95% Confidence/Credibility Range (Mean) | True Value |
| --- | --- | --- | --- |
| Gibbs Sampler No Errors | A | -0.16-0.26 (0.06) | 0.1 |
| Gibbs Sampler No Errors | B | -0.66-0.18 (-0.24) | 0.5 |
| Gibbs Sampler No Errors | C | 0.24-1.30 (0.76) | 2.6 |
| Gibbs Sampler With Errors | A | -0.03-0.64 (0.31) | 0.1 |
| Gibbs Sampler With Errors | B | -5.25-1.05 (-1.18) | 0.5 |
| Gibbs Sampler With Errors | C | 0.76-6.97 (2.76) | 2.6 |

**Model 4; 500 Patients**

| Fitting Technique | Parameter | 95% Confidence/Credibility Range (Mean) | True Value |
| --- | --- | --- | --- |
| Gibbs Sampler No Errors | A | -0.20-0.22 (0.01) | 0.1 |
| Gibbs Sampler No Errors | B | -0.54-0.31 (-0.12) | 0.6 |
| Gibbs Sampler No Errors | C | -0.08-0.94 (0.40) | 0.8 |
| Gibbs Sampler With Errors | A | -0.09-0.61 (0.24) | 0.1 |
| Gibbs Sampler With Errors | B | -3.38-2.20 (-0.54) | 0.6 |
| Gibbs Sampler With Errors | C | -0.10-5.75 (1.37) | 0.8 |

**Model 1; 1000 Patients**

| Fitting Technique | Parameter | 95% Confidence/Credibility Range (Mean) | True Value |
| --- | --- | --- | --- |
| Gibbs Sampler No Errors | A | -0.09-0.23 (0.07) | 0.2 |
| Gibbs Sampler No Errors | B | 0.03-0.67 (0.35) | 4 |
| Gibbs Sampler No Errors | C | 0.46-1.15 (0.78) | 3 |
| Gibbs Sampler With Errors | A | -0.02-0.43 (0.20) | 0.2 |
| Gibbs Sampler With Errors | B | 0.25-7.11 (2.60) | 4 |
| Gibbs Sampler With Errors | C | 1.49-8.03 (3.44) | 3 |

**Model 2; 1000 Patients**

| Fitting Technique | Parameter | 95% Confidence/Credibility Range (Mean) | True Value |
| --- | --- | --- | --- |
| Gibbs Sampler No Errors | A | 0.14-0.45 (0.29) | 0.5 |
| Gibbs Sampler No Errors | B | 0.01-0.63 (0.32) | 8 |
| Gibbs Sampler No Errors | C | 0.35-1.04 (0.68) | 3 |
| Gibbs Sampler With Errors | A | 0.29-0.77 (0.50) | 0.5 |
| Gibbs Sampler With Errors | B | 0.03-7.00 (2.37) | 8 |
| Gibbs Sampler With Errors | C | 1.11-8.03 (3.35) | 3 |

**Model 3; 1000 Patients**

| Fitting Technique | Parameter | 95% Confidence/Credibility Range (Mean) | True Value |
| --- | --- | --- | --- |
| Gibbs Sampler No Errors | A | -0.25-0.06 (-0.09) | 0.1 |
| Gibbs Sampler No Errors | B | -0.30-0.32 (0.00) | 0.5 |
| Gibbs Sampler No Errors | C | 0.60-1.28 (0.94) | 2.6 |
| Gibbs Sampler With Errors | A | -0.20-0.29 (0.05) | 0.1 |
| Gibbs Sampler With Errors | B | -1.57-2.31 (-0.01) | 0.5 |
| Gibbs Sampler With Errors | C | 1.75-8.70 (3.89) | 2.6 |

**Model 4; 1000 Patients**

| Fitting Technique | Parameter | 95% Confidence/Credibility Range (Mean) | True Value |
| --- | --- | --- | --- |
| Gibbs Sampler No Errors | A | -0.31--0.00 (-0.16) | 0.1 |
| Gibbs Sampler No Errors | B | -0.21-0.38 (0.09) | 0.6 |
| Gibbs Sampler No Errors | C | 0.09-0.72 (0.41) | 0.8 |
| Gibbs Sampler With Errors | A | -0.21-0.26 (0.03) | 0.1 |
| Gibbs Sampler With Errors | B | -0.90-1.89 (0.31) | 0.6 |
| Gibbs Sampler With Errors | C | 0.20-2.06 (0.98) | 0.8 |

**Model 1; 5000 Patients**

| Fitting Technique | Parameter | 95% Confidence/Credibility Range (Mean) | True Value |
| --- | --- | --- | --- |
| Gibbs Sampler No Errors | A | 0.05-0.19 (0.12) | 0.2 |
| Gibbs Sampler No Errors | B | 0.24-0.53 (0.38) | 4 |
| Gibbs Sampler No Errors | C | 0.65-0.97 (0.81) | 3 |
| Gibbs Sampler With Errors | A | 0.15-0.34 (0.24) | 0.2 |
| Gibbs Sampler With Errors | B | 1.51-7.22 (3.22) | 4 |
| Gibbs Sampler With Errors | C | 2.54-7.87 (4.27) | 3 |

**Model 2; 5000 Patients**

| Fitting Technique | Parameter | 95% Confidence/Credibility Range (Mean) | True Value |
| --- | --- | --- | --- |
| Gibbs Sampler No Errors | A | 0.26-0.39 (0.32) | 0.5 |
| Gibbs Sampler No Errors | B | 0.21-0.49 (0.34) | 8 |
| Gibbs Sampler No Errors | C | 0.56-0.90 (0.72) | 3 |
| Gibbs Sampler With Errors | A | 0.42-0.62 (0.52) | 0.5 |
| Gibbs Sampler With Errors | B | 1.34-7.53 (3.34) | 8 |
| Gibbs Sampler With Errors | C | 2.29-7.89 (4.06) | 3 |

**Model 3; 5000 Patients**

| Fitting Technique | Parameter | 95% Confidence/Credibility Range (Mean) | True Value |
| --- | --- | --- | --- |
| Gibbs Sampler No Errors | A | -0.10-0.03 (-0.03) | 0.1 |
| Gibbs Sampler No Errors | B | -0.02-0.25 (0.12) | 0.5 |
| Gibbs Sampler No Errors | C | 0.67-0.98 (0.82) | 2.6 |
| Gibbs Sampler With Errors | A | -0.03-0.19 (0.08) | 0.1 |
| Gibbs Sampler With Errors | B | 0.01-1.52 (0.68) | 0.5 |
| Gibbs Sampler With Errors | C | 2.09-7.19 (3.43) | 2.6 |

**Model 4; 5000 Patients**

| Fitting Technique | Parameter | 95% Confidence/Credibility Range (Mean) | True Value |
| --- | --- | --- | --- |
| Gibbs Sampler No Errors | A | -0.16--0.02 (-0.09) | 0.1 |
| Gibbs Sampler No Errors | B | 0.01-0.28 (0.15) | 0.6 |
| Gibbs Sampler No Errors | C | 0.25-0.55 (0.41) | 0.8 |
| Gibbs Sampler With Errors | A | -0.03-0.19 (0.08) | 0.1 |
| Gibbs Sampler With Errors | B | 0.07-2.17 (0.65) | 0.6 |
| Gibbs Sampler With Errors | C | 0.58-1.42 (1.00) | 0.8 |

**Model 1; 10000 Patients**

| Fitting Technique | Parameter | 95% Confidence/Credibility Range(Mean) | True Value |
| --- | --- | --- | --- |
| Gibbs Sampler No Errors | A | 0.05-0.15 (0.10) | 0.2 |
| Gibbs Sampler No Errors | B | 0.27-0.47 (0.37) | 4 |
| Gibbs Sampler No Errors | C | 0.61-0.84 (0.72) | 3 |
| Gibbs Sampler With Errors | A | 0.14-0.28 (0.21) | 0.2 |
| Gibbs Sampler With Errors | B | 1.67-6.83 (2.96) | 4 |
| Gibbs Sampler With Errors | C | 2.16-5.43 (3.18) | 3 |

**Model 2; 10000 Patients**

| Fitting Technique | Parameter | 95% Confidence/Credibility Range (Mean) | True Value |
| --- | --- | --- | --- |
| Gibbs Sampler No Errors | A | 0.27-0.36 (0.31) | 0.5 |
| Gibbs Sampler No Errors | B | 0.21-0.41 (0.32) | 8 |
| Gibbs Sampler No Errors | C | 0.54-0.78 (0.66) | 3 |
| Gibbs Sampler With Errors | A | 0.43-0.57 (0.50) | 0.5 |
| Gibbs Sampler With Errors | B | 1.53-7.36 (2.97) | 8 |
| Gibbs Sampler With Errors | C | 2.08-7.28 (3.35) | 3 |

**Model 3; 10000 Patients**

| Fitting Technique | Parameter | 95% Confidence/Credibility Range (Mean) | True Value |
| --- | --- | --- | --- |
| Gibbs Sampler No Errors | A | -0.07-0.02 (-0.02) | 0.1 |
| Gibbs Sampler No Errors | B | -0.05-0.14 (0.04) | 0.5 |
| Gibbs Sampler No Errors | C | 0.62-0.84 (0.73) | 2.6 |
| Gibbs Sampler With Errors | A | 0.06-0.21 (0.14) | 0.1 |
| Gibbs Sampler With Errors | B | -0.20-0.61 (0.22) | 0.5 |
| Gibbs Sampler With Errors | C | 1.75-3.09 (2.29) | 2.6 |

**Model 4; 10000 Patients**

| Fitting Technique | Parameter | 95% Confidence/Credibility Range (Mean) | True Value |
| --- | --- | --- | --- |
| Gibbs Sampler No Errors | A | -0.12--0.02 (-0.07) | 0.1 |
| Gibbs Sampler No Errors | B | -0.04-0.15 (0.06) | 0.6 |
| Gibbs Sampler No Errors | C | 0.15-0.36 (0.26) | 0.8 |
| Gibbs Sampler With Errors | A | 0.07-0.22 (0.15) | 0.1 |
| Gibbs Sampler With Errors | B | -0.12-0.66 (0.26) | 0.6 |
| Gibbs Sampler With Errors | C | 0.34-0.87 (0.60) | 0.8 |

**Model 1; 20000 Patients**

| Fitting Technique | Parameter | 95% Confidence/Credibility Range (Mean) | True Value |
| --- | --- | --- | --- |
| Gibbs Sampler No Errors | A | 0.08-0.14 (0.11) | 0.2 |
| Gibbs Sampler No Errors | B | 0.39-0.53 (0.46) | 4 |
| Gibbs Sampler No Errors | C | 0.63-0.79 (0.70) | 3 |
| Gibbs Sampler With Errors | A | 0.18-0.28 (0.24) | 0.2 |
| Gibbs Sampler With Errors | B | 2.98-8.63 (4.93) | 4 |
| Gibbs Sampler With Errors | C | 2.08-4.17 (2.72) | 3 |

**Model 2; 20000 Patients**

| Fitting Technique | Parameter | 95% Confidence/Credibility Range (Mean) | True Value |
| --- | --- | --- | --- |
| Gibbs Sampler No Errors | A | 0.30-0.37 (0.34) | 0.5 |
| Gibbs Sampler No Errors | B | 0.34-0.49 (0.42) | 8 |
| Gibbs Sampler No Errors | C | 0.55-0.72 (0.63) | 3 |
| Gibbs Sampler With Errors | A | 0.50-0.59 (0.54) | 0.5 |
| Gibbs Sampler With Errors | B | 2.89-8.46 (5.04) | 8 |
| Gibbs Sampler With Errors | C | 2.14-5.82 (2.90) | 3 |

**Model 3; 20000 Patients**

| Fitting Technique | Parameter | 95% Confidence/Credibility Range (Mean) | True Value |
| --- | --- | --- | --- |
| Gibbs Sampler No Errors | A | -0.07-0.02 (-0.02) | 0.1 |
| Gibbs Sampler No Errors | B | -0.05-0.14 (0.04) | 0.5 |
| Gibbs Sampler No Errors | C | 0.62-0.84 (0.73) | 2.6 |
| Gibbs Sampler With Errors | A | 0.07-0.18 (0.12) | 0.1 |
| Gibbs Sampler With Errors | B | 0.34-1.00 (0.66) | 0.5 |
| Gibbs Sampler With Errors | C | 1.94-2.90 (2.33) | 2.6 |

**Model 4; 20000 Patients**

| Fitting Technique | Parameter | 95% Confidence/Credibility Range (Mean) | True Value |
| --- | --- | --- | --- |
| Gibbs Sampler No Errors | A | -0.12--0.02 (-0.07) | 0.1 |
| Gibbs Sampler No Errors | B | -0.04-0.15 (0.06) | 0.6 |
| Gibbs Sampler No Errors | C | 0.15-0.36 (0.26) | 0.8 |
| Gibbs Sampler With Errors | A | 0.07-0.22 (0.15) | 0.1 |
| Gibbs Sampler With Errors | B | -0.12-0.66 (0.26) | 0.6 |
| Gibbs Sampler With Errors | C | 0.34-0.87 (0.60) | 0.8 |
